# The Cost-Effectiveness of an Internet Intervention to Facilitate Mental Health Help-Seeking by Young Adults: Randomized Controlled Trial

**DOI:** 10.2196/13065

**Published:** 2019-07-22

**Authors:** Long Khanh-Dao Le, Lena Sanci, Mary Lou Chatterton, Sylvia Kauer, Kerrie Buhagiar, Cathrine Mihalopoulos

**Affiliations:** 1 Deakin Health Economics, Institute for Health Transformation School of Health and Social Development Deakin University Burwood Australia; 2 Department of General Practice University of Melbourne Melbourne Australia; 3 ReachOut Australia NSW Australia

**Keywords:** economic evaluation, cost effectiveness, mental health, help-seeking, internet intervention

## Abstract

**Background:**

Little empirical evidence is available to support the effectiveness and cost-effectiveness of internet interventions to increase help-seeking behavior for mental health in young adults.

**Objective:**

The aim of this study was to evaluate the cost-effectiveness of a Web-based mental health help-seeking navigation tool (*Link*) in comparison with usual help-seeking strategies.

**Methods:**

A cost-utility analysis alongside the main randomized trial of *Link* was conducted from the Australian health care sector perspective. Young adults aged 18 to 25 years were randomized to the *Link* intervention (n=205) or usual care (n=208) with 1- and 3-month follow-ups. The primary outcome of this study was quality-adjusted life years (QALYs) measured by the assessment of quality of life–4D. Costs were calculated based on the self-reported resource use questionnaire and were reported in 2015 Australian dollars. Primary analyses were conducted as intention-to-treat and reported as incremental cost-effectiveness ratios. Completer analyses were conducted in a sensitivity analysis.

**Results:**

Significantly more QALYs were gained in the intervention group than the control group (0.15 vs 0.14; *P*<.001). The intervention was associated with significantly lower health professional consultation costs at 1-month follow-up (mean costs Aus $98 vs Aus $162; *P*<.05). Costs of hospital services were lower at 3 months in the intervention arm (mean costs Aus $47 vs Aus $101); however, there was insufficient sample size to detect a significant difference between the groups. There were no statistically significant differences in the total costs between the 2 arms. Relative to the control group, those who received the intervention experienced 0.01 more QALYs (0.00-0.02) and had lower total health sector costs of Aus −$81 (Aus −$348 to Aus $186) over 3 months. The intervention was found to be more effective and less costly compared with usual help-seeking strategies. The intervention was 100% likely to be cost-effective below a willingness-to-pay value-for-money threshold of Aus $28,033 per QALY. Results were robust in the sensitivity analysis.

**Conclusions:**

Our study found that the online youth mental health help-seeking Web service is a cost-effective intervention for young people aged 18 to 25 years compared with usual search strategies. Further research is required to confirm these results.

**Trial Registration:**

Australian New Zealand Clinical Trials Registry ACTRN12614001223628; https://www.anzctr.org.au /Trial/Registration/TrialReview.aspx?id=366731

## Introduction

### Background

Mental and substance use disorders are a leading cause of disability in children and young adults worldwide [1], making these diagnoses a significant public health concern. Mental disorders were also associated with substantial economic burden with an estimated total cost of Aus $12.7 billion annually within the Australian context [2]. Despite the significant effect of these conditions in young people, which may continue into adulthood, only 23.3% of young adults (aged 16-24 years) with a 12-month diagnosis of a mental disorder in Australia sought professional treatment for mental health problems [3].

Barriers to help-seeking and treatment for young people include stigma [4-7], embarrassment [5], poor mental health literacy [5,7], lack of knowledge about appropriate mental health services [6-8], and a preference for self-reliance [5,6] in addition to geographic barriers for those living in rural settings with limited access to resources [9,10]. E-mental health interventions delivered through internet or mobile phone technology show promise [11]; however, little empirical evidence is available to support the effectiveness and cost-effectiveness of these interventions to increase help-seeking behavior [12].

### Objective

To address these concerns, a randomized controlled trial (RCT) was conducted to evaluate the effectiveness and cost-effectiveness of a brief, internet-based, mental health help-seeking intervention, called *Link* compared with usual help-seeking strategies for young adults. The current analysis sought to answer whether an online help-seeking intervention for young adults was cost-effective compared with usual search practices from a health care sector perspective (defined as health care government expenditure plus health care out-of-pocket expenditure) within a 3-month follow-up.

## Methods

### Approval and Ethical Considerations

Ethics approval was obtained from the University of Melbourne Human Research Ethics Committee, reference #1341063.4, and Deakin University Human Research Ethics Committee, reference #2015-320. All participants consented to take part in this study via an online consent form.

### Study Design and Participants

This economic evaluation was conducted alongside the RCT. The study adheres to the Consolidated Health Economic Evaluation Reporting Standards Statement (CHEERS) checklist [13] (Multimedia Appendix 1).

The study was conducted entirely online. Participants were recruited by electronic direct mail, social media, online advertising, and snowballing, where participants were asked to share the link on the Facebook page with friends and family. Interested participants were directed from a link in the advertisements to the study website where they were provided with more information and a consenting procedure if meeting the eligibility criteria of being aged between 18 and 25 years and residing in Australia. Eligible participants provided informed consent by acknowledging that they had read the information statement by clicking a box, then clicking a separate box to indicate that they consented to participate in the Link Research Project. They then registered for the trial using their email address and a self-generated password. Immediately following registration, all participants completed the baseline survey sent through email including demographic information and the Kessler-10 (K10) measure of psychological distress. Participants were then stratified by responses on gender (male or female) and severity of psychological distress (K10>20), then randomized into parallel groups consisting of the intervention group (*Link*) or control group (usual search strategies) using a random allocation sequence generated internally by the QuON computer software [14]. Randomization was stratified by gender (male and female) and psychological distress (K10 score<20 and K10 score≥20) using random sequences of block sizes of 4, 6, or 8 within each stratum and an allocation ratio of 1:1. Online surveys were completed by all participants at baseline, postintervention, and 3-month follow-up. Survey measures included the positive affect and negative affect scale, barriers to adolescent help-seeking, stages of change questionnaire, K10, general help-seeking questionnaire, assessment of quality of life (AQoL)–4D, client satisfaction questionnaire, and the health service use questionnaire. Researchers and statisticians involved in the data analysis were blind to the allocation of participants until after data analysis was completed. Further information related to the trial can be found in the paper reporting the primary trial outcomes [15].

### Intervention Descriptions

#### Intervention Arm

The *Link* intervention is an online Web-based mental health help-seeking tool designed to guide young adults to appropriate online and offline sources of mental health information and care. The *Link* design is underpinned by the theory of planned behavior [16] and the Help-Seeking Model [17]. The functionality of *Link* operationalizes the elements of these theories (attitudes toward help-seeking, subjective norms, perceived control of help-seeking, and intentions to seek help) toward encouraging help-seeking behavior [18]. In brief, *Link* has a 4-step process where (1) users select symptoms they experience, (2) rate how much they are affected by them, (3) choose their preferred way to receive help (face-to-face, online information, telephone, and online chat), and then (4) finally, click on service options presented by the program for more information on how to seek help within that service, including expected costs and website links or online directories. The feasibility of *Link* was trialed previously and found to be acceptable to young people [19].

#### Control Arm: Usual Search Strategies

The control condition instructed the young adult participants to use their typical strategies to seek help both online and offline such as using internet search engines and face-to-face or phone services.

### Outcome Measures

#### Health-Related Quality of Life

The AQoL-4D was used to measure health-related quality of life [20]. Originally developed as a generic multiattribute utility instrument designed for the evaluation of public health interventions including mental health [20], it originally consisted of 15 items spread out into 5 dimensions measuring illness, independent living, social relationships, physical senses, and psychological well-being. However, the illness subscale was not used in the scoring [20]. The AQoL-4D scoring algorithm, based on the multiattribute utility theory, weighs the items and then applies a multiplicative model to obtain an index, which is transformed into a utility scale [20]. Quality-adjusted life years (QALYs) were calculated over the time horizon of the study using the area under the curve method [21].

#### Costs

This economic evaluation adopted a health sector perspective, which included health care costs paid by the government and out-of-pocket costs paid by patients. All costs were expressed as 2015 Australian Dollars. No discount rate was applied because the time horizon of the study was 3 months.

##### Intervention Costs

**Figure 1:**
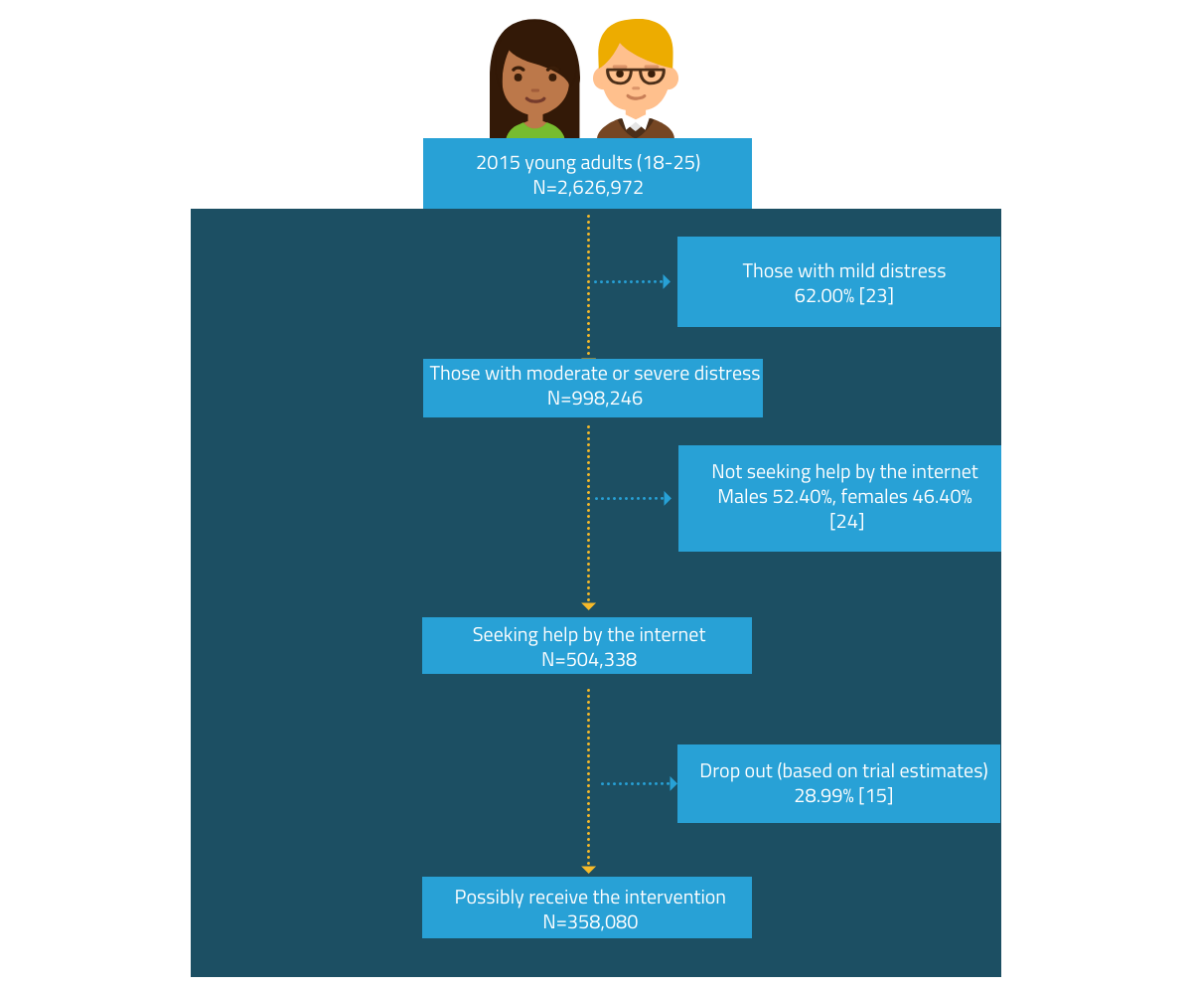
Estimation of population eligibility for the Link intervention.

Intervention costs comprised the intervention development costs and maintenance costs. Development costs were estimated from the details provided by the research team and included the planning, development, and production stages of the *Link* platform. The total projected cost for *Link* was Aus $1.74 million. The maintenance cost of the *Link* intervention included the time cost of 2 information technology staff (1 senior and 1 junior staff), in addition to the time cost of staff to update content and equipment costs. The total maintenance cost for *Link* was Aus $29,803 per year (or equivalent to Aus $2484 per month). To not overestimate the per-person costs (by assigning them only to trial participants), we estimated the number of people who are likely to receive the intervention when implemented within the Australian population using assumptions based on the published literature. The intervention pathway starts with young adults aged 18 to 25 years in the 2015 Australian population [22]. Despite no restriction of the intervention for young adults, we conservatively assumed that those with moderate or high mental health distress (measured by K10) are likely to have an interest in help-seeking for mental health problems [23]. For those people, approximately half were assumed to seek help through the internet based on the Mission Australia Youth Survey [24]. Furthermore, we also assumed a 29% dropout based on the dropout rate of this trial [15]. As a result, approximately 14% of Australian young adults were assumed to use the *Link* intervention (Figure 1).

This resulted in the average development cost per person for the *Link* program estimated at Aus $5.59, and the total average maintenance cost was estimated at Aus $0.04 per person per month. Therefore, the total intervention costs per person for the 3-month follow-up were estimated at approximately Aus $5.84.

##### Health Care Utilization Costs

Health care utilization was self-reported by participants at 1 and 3 months, retrospectively, using a resource use questionnaire (RUQ). The RUQ comprised questions on relevant health care services (eg, general practitioner [GP], psychologist, and/or mental health specialists or health experts), including the frequency of visits, payment methods (ie, out‐of‐pocket payments), outpatient care services (ie, nonadmitted hospital-based services), inpatient admissions, and medications. The different versions of the RUQ have been used in other trials in mental health [25]. The costs were calculated by multiplying the reported number of contacts by standard Australian unit costs. Unit costs for consultations (ie, GP, psychologist, psychiatrist, and allied health professionals) were sourced from the 2014 Medicare Benefit Schedule Book [22] and presented in Multimedia Appendix 2. Unit costs for medications adopted a weighted average of all available products containing the relevant active ingredient sourced from 2014 Pharmaceutical Benefit Schedule reports [26]. Hospital stays were costed using public sector average cost per separation through the Independent Hospital Pricing Authority, based on Australian Refined Diagnostic Related Group (AR-DRG) [27]. The specific AR-DRGs (for mental health symptoms) were chosen based on the self-reported reason and duration of stay.

The out-of-pocket costs reported in the RUQ for each service were considered in the health sector perspective. If the reported amount for a community-based health contact was outside of a plausible range, the maximum of out-of-pocket cost of Aus $447 was used based on the recommendation of the Australian Psychological Society [28]. For those who did not report out-of-pocket costs, we assumed that no out-of-pocket costs were incurred.

### Statistical Analysis

The primary analysis was performed using an intention-to-treat approach. All participants who were randomized were included in the analysis, and missing data were handled by multiple imputation by chained equations using predictive mean matching. The data were assumed to be missing at random by testing through a series of logistic regression analyses comparing participants’ characteristics for those with and without missing endpoint data. At 1- and 3-month follow-ups, approximately 30% of participants had dropped out or did not complete the survey (29% in the intervention group vs 31% in control group). However, the maximum percentage of missing QALY and cost data was 40%. Thus, to ensure efficient and reproducible estimates, a total of 40 imputations were completed [29,30]. The estimates obtained from each imputed dataset were combined using Rubin’s rules to generate an overall mean estimate of QALYs and costs. Rubin’s rules ensure that the standard error reflects the variability within and across imputations.

General linear models (GLMs) were used to evaluate differences between group on total QALYs and total health sector costs. For the GLMs, a modified Park test was used to identify the appropriate *family*, whereas Pregibon link test, Pearson correlation test, and modified Hosmer-Lemeshow test were adopted to identify the *link function* [21]. GLM with log link and Gaussian family was conducted for QALYs. Given the large proportion of zero costs, 2-part models were used to evaluate the difference in components of the total costs including consultations, hospital, and medication costs between intervention and control groups as recommended in the literature [21]. We first modeled the probability that a person has any health care expenditures with a logit model using the full sample. Then we estimated a GLM on the subset of people who have any expenditures. The 2-part model allows for separate investigation of the effect of covariates on the extensive margin (logit model, if any expenditures) and on the intensive margin (GLM, amount of expenditures if any) [31,32]. GLM using log link and gamma family was used for cost variables as recommended by the International Society for Pharmacoeconomics and Outcome Research guidelines [33]. All regression analyses were adjusted by the utility scores at baseline, gender (male and female), baseline K10 scores, and the use of online searches for mental health services in the 2 weeks before study entry. The incremental difference in costs and QALYs between groups was estimated based on the 3-month data using seemingly unrelated regression model, combining estimates of mean coefficients and the covariance matrix as per Rubin’s rules [34]. The regression coefficient on the treatment variable in the cost and QALY equations represents the incremental differences in costs and QALYs, respectively. The incremental cost-effectiveness ratio (ICER) was calculated as the ratio of these coefficients.

The bias-corrected CIs around the ICER were reported based on 3000 bootstrap simulations. The bootstrapped data were also plotted on a cost-effectiveness plane [35]. The threshold willingness-to-pay of Aus $28,033 per QALY gained was used to determine cost-effectiveness because this reflects the opportunity costs of decisions to publicly fund new health technologies in Australia [36]. In addition, a cost-effectiveness acceptability curve was constructed by calculating the probability of the intervention being cost-effective at different values of willingness-to-pay [37]. The probability of cost-effectiveness was estimated from combining mean coefficients and the covariance matrix from the seemingly unrelated regression model. The validity of this approach relies on the multivariate normality of the group-specific mean costs and QALYs [34]. This is appropriate with a sufficient sample size even when individual costs and QALYs are skewed [34,38].

Sensitivity analyses included a complete case analysis in which only participants who completed 1- and 3-month follow-ups were included. In addition, the development costs were varied to reflect different proportions of the population receiving the intervention if it was implemented in Australia. In particular, the proportion of people who would receive the intervention was varied from 2% to 17% of the Australian population.

All analyses were undertaken using Stata SE version 15.

## Results

### Overview

A total of 413 participants were randomized, with 205 allocated to *Link* and 208 allocated to the control group. Additional details regarding the study flow and Consort diagram are reported elsewhere [15]. The overall attrition rates were similar between the 2 study groups (71% *Link* vs 69% control group). Baseline characteristics were similar between the groups (Table 1), except a significantly greater proportion of participants in the intervention group carried out an online search of mental health services in the 2 weeks before randomization compared with the control group (38.5% vs 26%, *P*<.01).

**Table 1:** Baseline characteristics of the study population.

Characteristics		*Link* intervention (n=205)	Control (n=208)
**Gender, n (%)**			
	Female	171 (83.4)	173 (83.2)
	Other^a^	3 (1.5)	4 (1.9)
**Education, n (%)**			
	Completed secondary school	104 (50.7)	99 (47.6)
	Higher education	90 (43.9)	95 (45.7)
**Working status, n (%)^b^**			
	Yes	107 (52.2)	117 (56.3)
**Absent study days, n (%)**			
	Yes	58 (28.3)	71 (34.1)
**K10^c^ categories, n (%)**			
	Mild	28 (13.7)	39 (18.7)
	Moderate	38 (18.5)	26 (12.5)
	Severe	94 (45.8)	96 (46.2)
**Physical health self-rating, n (%)**			
	Some symptoms but no disease	87 (42.4)	85 (40.9)
	Minor illness	24 (11.7)	38 (18.3)
	Moderate to severe	24 (11.7)	24 (11.5)
**Mental health self-rating, n (%)**			
	Some symptoms but no disease	68 (33.2)	60 (28.9)
	Minor illness	34 (16.6)	48 (23.1)
	Moderate to severe	76 (37.1)	72 (34.6)
**Online mental health services search in the last 2 weeks, n (%)^d^**			
	Yes	79 (38.5)	54 (26.0)
Age (years), mean (SD)		20.89 (2.32)	21.30 (2.38)
Utility score, mean (SD)		0.56 (0.26)	0.56 (0.26)

^a^Other includes transgender and agender participants.

^b^Employment includes paid and unpaid (volunteer) workers.

^c^K10: Kessler-10.

^d^*P*=.01.

### Service Utilization

Use of health services is reported for baseline and 1- and 3-month follow-up periods in Table 2. GP services were the most commonly utilized services for both the groups at each time point. However, the only statistically significant service between intervention and control groups was online services at baseline. A subgroup analysis indicated that the *Link* intervention was associated with a lower number of lengthy health professional consultations; however, this difference did not reach statistical significance. For example, there were less people (2 vs 11) attending extensive GP consultations (duration over 40 min) in the intervention group at the 1-month follow-up compared with those who used usual search strategies.

### Outcomes

The estimated mean AQoL-4D utility values and QALYs for the intervention and control groups over the 3-month follow-up are presented in Table 3. The utility values increased over time for the intervention group but not for the control group. At the 3-month follow-up, the estimated mean utility value for the intervention group was significantly greater than for the control group (0.63 vs 0.56, *P*<.001). Similarly, there was a statistically significant difference in QALYs at the 3-month follow-up between the groups, which favored the intervention group (0.103 vs 0.093, *P*=.01).

**Table 2:** Health service uses at baseline and 1-month and 3-month follow-ups.

Service type^a^	Baseline, n (%)		1 month, n (%)			3 months, n (%)		
	Intervention	Control		Intervention	Control		Intervention	Control
General practitioner	135 (65.9)	128 (61.5)		51 (24.9)	53 (25.5)		58 (28.3)	47 (22.6)
Psychologist	47 (22.9)	56 (26.9)		21 (10.2)	22 (10.6)		24 (11.7)	22 (10.6)
Psychiatrist	18 (8.8)	27 (13.0)		6 (2.9)	9 (4.3)		5 (2.4)	7 (3.4)
Headspace	23 (11.2)	22 (10.6)		14 (6.8)	15 (7.2)		21 (10.2)	11 (5.3)
Other service	16 (7.8)	12 (5.8)		14 (6.8)	9 (4.3)		13 (6.3)	7 (3.4)
Online services	79 (38.5)^b^	54 (26.0)^b^		52 (25.3)	50 (24.0)		38 (18.5)	36 (17.3)
Medication	44 (21.5)	56 (26.9)		19 (9.3)	24 (11.5)		20 (9.8)	22 (10.6)
Hospital	26 (12.7)	24 (11.5)		2 (1.0)	6 (2.9)		3 (1.5)	4 (1.9)
No services used	55 (26.8)	57 (27.4)		52 (25.3)	46 (22.1)		49 (23.9)	57 (27.4)

^a^Subcategories are not mutually exclusive.

^b^*P*=.01.

**Table 3:** Mean costs per participant (in Aus $) by condition cumulative over the 1- or 3-month follow-up period (based on intention-to-treat sample, N=403).

Costs	1-month follow-up			3-month follow-up		
	Intervention, mean (95% CI), Aus $	Control, mean (95% CI), Aus $	*P* value	Intervention, mean (95% CI), Aus $	Control, mean (95% CI), Aus $	*P* value
Consultation costs	98 (73-123)	161 (103-220)	.01	214 (148-281)	206 (139-272)	.12
Hospital costs^a^	35 (0-94)	10 (0-19)	—^b^	46 (0-131)	107 (0-305)	—
Medication costs	7 (3-12)	7 (4-10)	.29	16 (6-25)	11 (6-16)	.05
Total costs (health care perspective)	145 (75-214)	178 (119-237)	.13	280 (168-392)	323 (106-540)	.64
Utility	0.60 (0.56-0.64)	0.55 (0.51-0.59)	.17	0.64 (0.60-0.68)	0.56 (0.52-0.60)	.003
Quality-adjusted life years	0.049 (0.046-0.051)	0.047 (0.044-0.049)	.37	0.103 (0.097-0.109)	0.093 (0.087-0.099)	.01

^a^Including inpatient and outpatient hospital costs.

^b^Insufficient observations for the 2-part model.

### Cost

As shown in Table 3, the average consultation costs at the 1-month follow-up and medication costs at the 3-month follow-up in the *Link* group were statistically significantly higher than those in the control group. No statistically significant differences for other cost categories at any other time points were found. The average total health sector costs for the intervention group were lower than the control group at 1-month and 3-month follow-ups. However, these differences were not statistically significantly different at both follow-up time points. The details of 2-part model results for medication and consultation cost are presented in Multimedia Appendix 3.

### Cost-Effectiveness

The results of the incremental analysis suggest that the *Link* intervention was associated with significantly higher utility-based quality of life than the control condition (mean difference 0.01, 95% CI 0.00-0.02). Furthermore, the *Link* intervention was also associated with lower costs (mean difference −81, 95% CI −348 to 186) compared with the control group; however, this difference did not reach statistical significance (Table 4). An intention-to-treat analysis indicated that the *Link* intervention was dominant (ie, more effective and less costly) compared with usual search strategies (95% CI dominant to Aus $11,867 per QALY).

**Figure 2:**
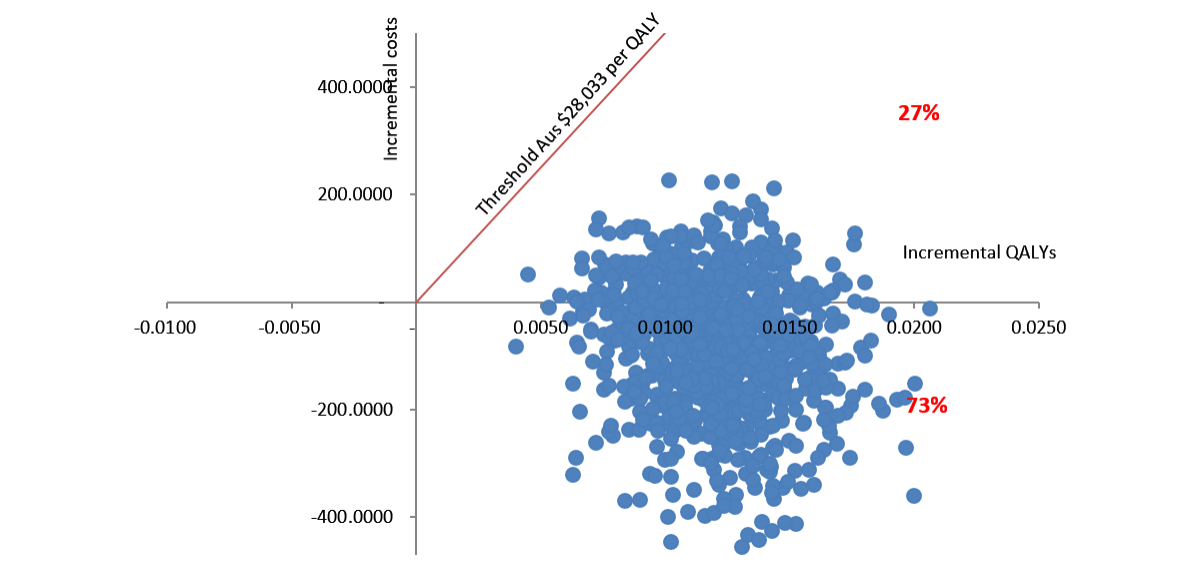
Cost-effectiveness plane of 3000 replicates of the incremental cost-effectiveness ratio—intent-to-treat analysis. QALY: quality-adjusted life year.

**Figure 3:**
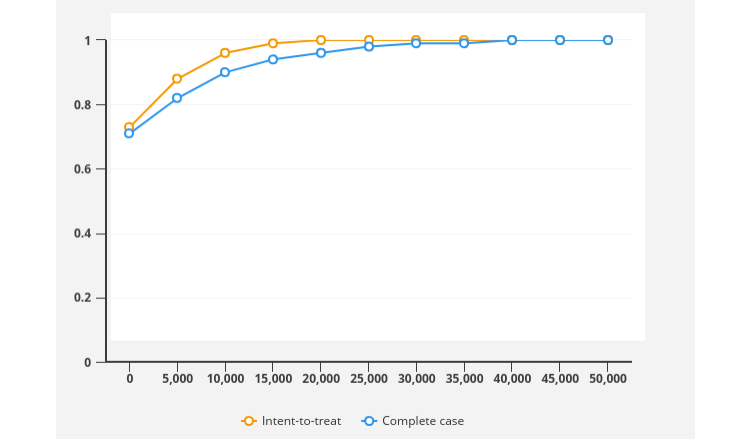
Cost-effectiveness acceptability curves for intent-to-treat and complete case analysis.

A probabilistic analysis showed that 100% of uncertainty iterations of the ICER fell below the threshold of Aus $28,303 per QALY gained, and 73% of iterations fell in the dominant quadrant of the cost-effectiveness plane (ie, more effective and less costly, Figure 2). The cost-effectiveness acceptability curve indicated that the *Link* intervention had a 95% probability of being cost-effective as long as the threshold of willingness-to-pay is over Aus $10,000 per QALY gains (Figure 3).

**Table 4:** Results of primary and sensitivity analyses (based on 3000 bootstrap simulations).

Analysis		Incremental costs, Aus $ (95% CI)	Incremental effects, quality-adjusted life year (95% CI)	ICER^a^, mean (95% CI)	Distribution over the ICER plane (%)			
					NE^b^	NW^b^	SE^b^	SW^b^
**Primary analysis**								
	Intention-to-treat analysis	−79 (−342 to 134)	0.01 (0.01 to 0.02)	Dominant (dominant to Aus $11,928)	27	—^c^	73	—
	Complete case analysis	−130 (−590 to 226)	0.01 (0.00 to 0.02)	Dominant (dominant to Aus $24,529)	29	—	71	—
**Sensitivity analysis**								
	Dropout rate 10% (cover 17% population); cost development per case: Aus $3.82	−85 (−363 to 134)	0.01 (0.00 to 0.02)	Dominant (dominant to Aus $13,035)	25	—	75	—
	Dropout rate 90% (cover 2% population); cost development per case: Aus $34.40	−50 (−319 to 159)	0.01 (0.00 to 0.02)	Dominant (dominant to Aus $14,564)	37	—	63	—

^a^ICER: incremental cost-effectiveness ratio, based on 3000 bootstrap simulation.

^b^In the northeast (NE) quadrant, the intervention is cost-effective if the ICER falls under the specified value-for-money criterion because the intervention is more effective and costlier than the comparator. In the southeast (SE) quadrant, the intervention is less costly and more effective than the comparator (ie, dominant); therefore, the intervention is likely to be excellent for value-for-money. In the southwest (SW) quadrant, the intervention is less costly and less effective; therefore, the decision to adopt the intervention may be based on decision-makers willingness to accept some health loss relative to cost-saving. Finally, in the northwest (NW) quadrant, the results show that the intervention is associated with greater costs but less health gain, therefore, not a good option to adopt.

^c^Not applicable.

### Sensitivity Analyses

The results for the intention-to-treat (using multiple imputation) concur with those for the complete case dataset, which show a similar pattern of greater effectiveness and less cost associated with the intervention group compared with the control group (Table 4 and Multimedia Appendix 4). The sensitivity analyses, which varied the proportion of people likely to receive the intervention from 2% to 17% of young adults aged 18 to 25 years, showed that results were very robust (Table 4).

## Discussion

### Principal Findings

This study was the first cost-utility analysis of an online intervention to increase mental health help-seeking for young adults (*Link*) compared with usual search strategies. Young people randomized to the Link intervention had significantly higher utility values and QALYs gained at 3 months compared with young people using their usual online search strategies. The online help-seeking intervention was also associated with lower average total costs from a health sector perspective although this did not reach statistical significance. The online help-seeking intervention was found to be a cost-effective treatment option compared with young adults’ current search strategies with a 73% probability that *Link* would be cost-saving and a 100% probability that it would be cost-effective using a willingness-to-pay threshold of Aus $28,033 per QALY gained. In fact, results suggest that even at a more modest Aus $10,000 per QALY value-for-money threshold, the intervention is still likely to be very cost-effective. The results were robust in the sensitivity analysis when complete case analysis was conducted, or the intervention costs were varied.

Interestingly, QALYs were improved in the *Link* group; however, the intervention did not appear to change resources used because quantities and costs of services were largely similar across the 2 groups. A possible explanation for this finding is that Link connected young people with higher quality, evidence-based targeted services compared with the treatment they might otherwise access.

### Comparison With Previous Work

The findings from this study are difficult to compare with other economic evaluations of internet-based interventions; as to our knowledge, this is the first study to evaluate the cost-effectiveness of an online resource to facilitate help-seeking behavior. Economic evaluations of internet interventions for mental health have been mostly focused on the treatment or prevention of mental disorders [39]. It is noteworthy that our study results are similar to economic evaluations that support the cost-effectiveness of guided internet educational and psychological interventions for the prevention and treatment of mental disorders [39,40]. More encouraging, our study indicated that the help-seeking intervention may be a very cost-effective, if not a cost-saving, option. Further research is required to confirm this result.

### Implications

Findings from our study showed that although there were no significant differences in terms of health care service use between the 2 groups, the *Link* intervention was significantly associated with lower health professional consultation costs at short-term follow-up (1 month). The reason might be that the intervention was associated with a reduction in the quantity of longer health professional (eg, GP or psychologist) consultations (duration over 40 min) than in the control group. Another important point is that the *Link* intervention was associated with lower hospitalization costs than the control at the 3-month follow-up, although the number of people who were hospitalized was not different. This might suggest a positive benefit of the *Link* intervention in reducing severity of mental health problems that require intensive treatments. However, these results did not reach significance, given that the sample sizes of these subgroups were relatively small. As noted above, these results may be explained by the quality of services being accessed via the *Link* platform. Further research with larger sample sizes and perhaps more evaluation of the type of care being accessed (in terms of quality) is needed.

This study, for the first time, raises the possibility that improving help-seeking not only assists young adults in accessing mental health care services but is also associated with quality of life improvements. More importantly, a Web-based mental health service navigation website (ie, *Link* platform) demonstrated a high probability of being cost-effective. The initial results from this study are certainly very promising and suggest that if access to the intervention was increased, this could result in significant health impacts and likely cost savings.

### Strengths and Limitations

This study has several strengths. First, this study used a cost-utility framework whereby outcomes are expressed as QALYs [37], thereby allowing results to be comparable with other economic evaluations and commonly used value-for-money thresholds. Second, this study adheres to the CHEERS checklist, which are quality reporting guidelines for economic evaluation [13]. Finally, a sensitivity analysis has been conducted to assess the robustness of the findings from the primary analysis.

In terms of limitations, these results do not include any costs beyond the health sector, which may underestimate the cost-effectiveness of the *Link* intervention. For example, the inclusion of productivity costs (absenteeism and presentism) may be associated with even more cost savings. The study was also limited by the relatively short time horizon (ie, 3 months) and the use of self-reported retrospective utilization of health care services and medication, potentially leading to recall bias. It is not clear whether this may have led to an over- or underestimation of resource use reporting, although any biases are likely to be the same in both groups. Further research using a broader societal perspective and longer follow-up is needed.

### Conclusions

In conclusion, the online help-seeking navigation website, *Link*, appears to provide a cost-effective and, possibly, cost-saving tool for young adults compared with the usual methods for seeking care. The intervention demonstrated a reduction in health care professional consultation costs at the 1-month follow-up and hospital costs at the 3-month follow-up. These results were robust in the sensitivity analysis. Further research to confirm these results could have important implications for increasing the accessibility of mental health care services for young adults.

## Appendix

Multimedia Appendix 1 Consolidated Health Economic Evaluation Reporting Standards Statement checklist.

Multimedia Appendix 2 Unit costs for health care consultation sourced from the 2014 Medicare Benefit Schedule Book.

Multimedia Appendix 3 Two-part models for consultation and medication costs (intent-to-treat analysis).

Multimedia Appendix 4 Mean costs per participant (in Aus $) by condition cumulative over the 1- or 3-month follow-up period (based on completer analysis).

Multimedia Appendix 5 CONSORT‐EHEALTH checklist (V 1.6.1).

## Abbreviations

AQoL: Assessment of Quality of Life
AR-DRG: Australian Refined Diagnostic Related Group
CHEERS: Consolidated Health Economic Evaluation Reporting Standards Statement
GLM: general linear model
GP: general practitioner
ICER: incremental cost-effectiveness ratio
K10: Kessler-10
NHMRC: National Health and Medical Research Council
QALY: quality-adjusted life year
RCT: randomized controlled trial
RUQ: resource use questionnaire
