# Supplementary material for: The Cost-Effectiveness of an Internet Intervention to Facilitate Mental Health Help-Seeking by Young Adults: Randomized Controlled Trial
Source: J Med Internet Res. 2019 Jul 22;21(7):e13065. doi: 10.2196/13065 (PMC6681639; doi:10.2196/13065)
Supplement: Multimedia Appendix 2 [file jmir_v21i7e13065_app2.docx]

Multimedia Appendix 2. Unit costs for health care consultation sourced from the 2014 Medicare Benefit Schedule Book (22).

| **Unit cost** | **Value (A$)** |
| --- | --- |
| **General Practitioner** |  |
| <20 minutes | 36.01 |
| 20 minutes to 40 minutes | 71.37 |
| >40 minutes | 109.83 |
| **Psychologist** |  |
| <60 minutes | 74.35 |
| >60 minutes | 87.94 |
| **Psychiatrist** |  |
| <15 minutes | 40.25 |
| 15 – 30 minutes | 79.61 |
| 30 – 45 minutes | 125.17 |
| >45 minutes | 201.9 |
| **Headspace service** |  |
| <15 minutes | 36.02 |
| 15-30 minutes | 71.49 |
| >30 minutes | 116.44 |
| **Other services** |  |
| <50 minutes | 58.60 |
| >50 minutes | 79.02 |
