# Supplementary material for: The Cost-Effectiveness of an Internet Intervention to Facilitate Mental Health Help-Seeking by Young Adults: Randomized Controlled Trial
Source: J Med Internet Res. 2019 Jul 22;21(7):e13065. doi: 10.2196/13065 (PMC6681639; doi:10.2196/13065)
Supplement: Multimedia Appendix 3 [file jmir_v21i7e13065_app3.docx]

Multimedia Appendix 3. Two-part Models for consultation and medication costs (intent-to-treat analysis)

|  | **1-month follow up** | |  | **3-month follow up** | |
| --- | --- | --- | --- | --- | --- |
|  | **Probability of cost** | **Total cost given cost>0** |  | **Probability of cost** | **Total cost given cost>0** |
|  | **Coefficient (SE)** | **Coefficient (SE)** |  | **Coefficient (SE)** | **Coefficient (SE)** |
| **Medication cost** |  |  |  |  |  |
| Intercept | -0.63 (1.08) | 3.20 (1.60)* |  | -0.16 (1.19) | 4.43 (1.71)* |
| Treatment arm (Link) | -0.25 (0.20) | 0.31 (0.29) |  | -0.23 (0.22) | 0.53 (0.26)* |
| Sex (female) | 0.37 (0.32) | 0.25 (0.43) |  | 0.58 (0.37) | -0.24 (0.66) |
| Age | 0.00 (0.04) | -0.01 (0.05) |  | -0.04 (0.04) | -0.03 (0.06) |
| Baseline utility | -1.59 (0.49)** | 0.55 (0.65) |  | -1.47 (0.51)** | 0.49 (0.80) |
| Baseline online service used (yes) | 0.24 (0.20) | -0.12 (0.23) |  | 0.24 (0.23) | 0.04 (0.26) |
| Baseline K10 score | 0.00 (0.02) | 0.01 (0.02) |  | 0.00 (001) | 0.02 (0.02) |
| **Consultation cost** |  |  |  |  |  |
| Intercept | 0.23 (0.91) | 4.61 (1.34)** |  | 0.09 (0.94) | 8.53 (0.94)*** |
| Treatment arm (Link) | -0.17 (0.16) | -0.45 (0.18)* |  | 0.21 (0.16) | -0.23 (0.15) |
| Sex (female) | 0.11 (0.22) | 0.25 (0.20) |  | -0.00 (0.19) | -0.18 (0.20) |
| Age | -0.02 (0.03) | 0.00 (0.04) |  | -0.01 (0.03) | -0.09 (0.03)* |
| Baseline utility | -0.61 (0.42) | -0.07 (0.47) |  | -0.78 (0.37)* | -1.34 (0.40)** |
| Baseline online service used (yes) | 0.23 (0.19) | 0.38 (0.20) |  | 0.15 (0.17) | 0.15 (0.17) |
| Baseline K10 score | 0.02 (0.01) | 0.01 (0.01) |  | 0.02 (0.01) | 0.01 (0.01) |

*P<0.05; **P<0.01; P<0.001; SE: standard error; K10: Kessler-10
