# Supplementary material for: The Cost-Effectiveness of an Internet Intervention to Facilitate Mental Health Help-Seeking by Young Adults: Randomized Controlled Trial
Source: J Med Internet Res. 2019 Jul 22;21(7):e13065. doi: 10.2196/13065 (PMC6681639; doi:10.2196/13065)
Supplement: Multimedia Appendix 4 [file jmir_v21i7e13065_app4.docx]

Multimedia Appendix 4. Mean costs per-participant (in AUD$) by condition cumulative over the one or three-month follow-up period (based on completer analysis)

|  | **One-month follow up** | | | **Three-month follow up** | | |
| --- | --- | --- | --- | --- | --- | --- |
|  | *Link* | Control | *P* value | *Link* | Control | *P* value |
| **Consultation** | 96 | 182 | *P* = 0.02 | 217 | 207 | *P* = 0.16 |
|  | (69, 123) | (106, 257) |  | (140, 293) | (137, 276) |  |
|  |  |  |  |  |  |  |
| **Hospital costs ^a^** | 35 | 11 | N/A^b^ | 68 | 164 | N/A^b^ |
|  | (0, 101) | (0, 22) |  | (0, 195) | (0, 475) |  |
|  |  |  |  |  |  |  |
| **Medication** | 8 | 6 | *P* = 0.19 | 17 | 10 | *P* = 0.07 |
|  | (2, 14) | (4, 9) |  | (5, 29) | (5, 15) |  |
|  |  |  |  |  |  |  |
| **Total costs (societal perspective)** | 156 | 194 | *P* = 0.30 | 333 | 387 | *P* = 0.77 |
|  | (81, 233) | (118, 271) |  | (174, 493) | (44, 729) |  |
|  |  |  |  |  |  |  |
| **Utility** | 0.60 | 0.55 | *P* = 0.08 | 0.64 | 0.57 | *P* < 0.001 |
|  | (0.56, 0.65) | (0.51, 0.60) |  | (0.59, 068) | (0.52, 0.62) |  |
|  |  |  |  |  |  |  |
| **QALYs** | 0.049 | 0.047 | *P* = 0.08 | 0.105 | 0.093 | *P* = 0.002 |
|  | (0.046, 0.053) | (0.043, 0.050) |  | (0.097, 0.112) | (0.085, 0.101) |  |
|  |  |  |  |  |  |  |

^a^ including inpatient and outpatient hospital costs

^b^ insufficient observations for two-part model.

QALYs: Quality adjusted-life years.
